# Swearing as a catalyst of technological development

**DOI:** 10.3389/fpsyg.2026.1792363

**Published:** 2026-03-30

**Authors:** Antonio Benítez-Burraco, Nicholas Washmuth

**Affiliations:** 1 Department of Spanish, Linguistics and Theory of Literature (Linguistics), Faculty of Philology, University of Seville, Seville, Spain; 2 Department of Psychology, The University of Alabama in Huntsville, Huntsville, AL, United States

**Keywords:** human self-domestication, language evolution, motor skills, swearing, technological change

## Introduction

Humans excel in our technological abilities, which have been linked to evolutionary changes in our mind, body (including the hands), social behavior, and even the environment (see Ambrose, 2001, or Foley and Lahr, 2003 for reviews). On an apparently different note, increasing evidence suggests that manual dexterity is potentiated by swearing. Indeed, swearing elicits many measurable biopsychosocial positive effects, like strengthened attention and memory, heightened autonomic arousal, or hypoalgesia (i.e., pain relief) (Stapleton et al., 2022; Washmuth et al., 2026). In this opinion paper, we will support the view that swearing behavior may have contributed to the advancement of human technology. In principle, it is emotionally neuter and significantly elaborated (i.e., lexically rich and grammatically complex) language that can be expected to support technological improvements, since it enables more efficient transmission of know-hows to others. Indeed, as societies evolve larger and more complex, the languages they speak usually gain more content words (Goulden et al., 1990; Bromham et al., 2015; Reali et al., 2018) and richer lexical taxonomies (Kay and Maffi, 1999; Lev-Ari, 2024), and their grammars become more complex and rule-dependent (Wray and Grace, 2007; Chen et al., 2024). Nonetheless, here we will argue that swear words, in spite of being structurally simpler and emotionally loaded, might have contributed, directly and indirectly, to improving our technological abilities. Directly, because of its positive biopsychosocial effects, particularly, on fine motor skills, as noted. Indirectly, because an emotionally alliviated state with decreased stress levels and reduced anxiety, as a result of swearing, enables one to engage in more efficient deep thinking, more productive work within a community, and more sophisticated linguistic behavior: not just richer language uses, but also more complex language structures, which largely result from a cultural mechanism (Tamariz and Kirby, 2016; Tamariz, 2017). We will frame our hypothesis within the self-domestication hypothesis of human evolution (henceforth, HSD).

## Human self-domestication: why we evolved to be less aggressive

Humans feature distinctive cognitive and behavioral traits that account for their evolutionary success. A recent view is that most aspects of the human phenotype reflect a domestication-like process in which ecological pressures and shifting social structures led to reduced reactive (i.e., automatic) aggression. In brief, factors like changes in our foraging ecology, climate deterioration, the colonization of new territories, and/or the advent of co-parenting, as childcare became more demanding because of our increasingly bigger brains, would have selected less aggressive individuals in our species (e.g., Pisor and Surbeck, 2019; Spikins et al., 2021; McCall, 2025). In turn, selection against reactive aggression would have triggered in humans the suite of features typically observed in domestic animals through a similar embryonic mechanism (Wilkins et al., 2014; see Sánchez-Villagra et al., 2016 for some clarifications). As a result, our social cognition became improved, our cooperation increased, and complex social networks emerged, which are all factors necessary for developing sophisticated cultures and, particularly, advanced technologies (see Hare, 2017, or Hare and Woods, 2020, for details).

## Language evolution under the effects of self-domestication: the role of swearing

HSD has been hypothesized to foster, specifically, the emergence of complex languages through neurocognitive and cultural mechanisms (see Thomas and Kirby, 2018; Raviv and Kirby, 2023; Benítez-Burraco, 2025 for general discussions). With regards to the former, the brain regions that control aggressive responses are functionally connected to, or even partially overlapping with, the areas which process language (Miller et al., 2008). As discussed in detail by Benítez-Burraco and Progovac (2021), increased control on aggression typically involves higher control (and a functional invasion) of subcortical areas by the cortex, but this can be expected to potentiate grammar processing as well. The reason is that the syntax network encompasses both subcortical regions (including the striatum, which is a core component of the procedural memory storing grammar rules) and parts of the cortex (where working memory is located) (Lieberman, 2000; Ullman, 2001; Ullman et al., 2020; Murphy et al., 2022). Regarding the cultural mechanisms, the lower levels of reactive aggression as we evolved self-domesticated seemingly facilitated the establishment of more diverse, frequent, and prolonged contacts between people, which are all factors that make languages more complex via a cultural mechanism (Tamariz and Kirby, 2016; Tamariz, 2017; Benítez-Burraco, 2025).

A recent detailed evolutionary model for language under the effect of HSD (Benítez-Burraco and Progovac, 2020) proposes four specific stages in the sophistication of language as humans became increasingly self-domesticated. The first stage corresponds to languages consisting of just single-word utterances mostly aimed to convey emotions, as in commands, threats, or exclamations. The last stage corresponds to the sort of full-fledged languages spoken by modern complex societies (like Chinese or English), with expanded vocabularies and sophisticated syntaxes, which, as noted, seem very suited for exchanging complex information and know-hows with strangers, and ultimately, for supporting advanced technology. Importantly for our concerns here, according to this model, once HSD started to increase, first rudimentary two-slot grammars emerged, enabling humans to combine single word utterances in a pair-wise fashion. In the model, such two-word combinations are hypothesized to have been used not only for expressing predications about the world around (“see deer”) or commanding more efficiently on others (“look man”), but for creating derogatory compound expressions (“killjoy”). Hurling insults at others might have thus replaced direct physical aggression, which is more harmful, as the main way of settling conflicts (Progovac and Benítez-Burraco, 2019). Actually, it was Freud (1893) who famously said that the man who first flung a word of abuse at his enemy instead of a spear was the founder of civilization. In other words, as HSD increased, “reactive language” would have replaced “reactive aggression”. At the same time, because of the (partially) common brain mechanisms involved, as characterized above, creating and using this type of derogatory compounds can be hypothesized to have reinforced our trend toward a more prosocial behavior (see Progovac and Benítez-Burraco, 2019 for details).

## Swearing and motor skills

Ontogenetically, a positive relationship exists between motor abilities, social cognition and social interactions, and language skills (Leonard and Hill, 2014). For instance, motor improvements by the child typically result in more frequent and richer interactions with other individuals, this in turn potentiating her language abilities (Iverson, 2022). Neurobiological determinants can contribute as well to such a positive feedback loop between motor and language development. As noted above, procedural memory is crucially involved in the processing of syntactic rules, and more generally, the compositional, automated, rule-governed aspects of language (Ullman, 2004, 2016). But procedural memory is also necessary for other rule-governed behaviors, including most of the motor behaviors supporting human technology, like toolmaking (Muller et al., 2023). The same can be said of our working memory, which is necessary not only for processing complex language, but also for supporting the sort of complex motor behaviors found in human technology. Not surprisingly, the increasing sophistication of lithic technologies during Prehistory has been linked to the potentiation of our working memory (Wynn and Coolidge, 2004).

Focusing on derogatory language, recent experimental evidence supports a link between swearing and motor skills, too. Accordingly, Washmuth et al. (2026) found that repeating a swear word immediately prior to a manual dexterity task enhanced performance compared with repeating a neutral word. Such swearing-induced performance improvements were observed across different tasks, samples, and testing conditions (Stephens et al., 2025; Washmuth et al., 2026). These findings are consistent with prior, more anecdotal evidence. For instance, surgeons, known for their high-level motor skills, have been observed swearing in the operating room and during other fine-motor tasks (Palazzo and Warner, 1999; Joseph et al., 2024). These direct effects of swearing on motor performance reinforce the view of some shared neurobiological mechanism for language and motor behavior of the sort hypothesized above. Still, in addition to these direct effects, swearing can be expected to promote motor improvements in more indirect ways. For instance, repeating a swear word has been found to enhance motor performance by shifting people into a more disinhibited, goal-focused, self-confident psychological state (Stephens et al., 2025). More generally, swearing reduces stress, provides catharsis, and facilitates emotional release (Vingerhoets et al., 2013; Stapleton et al., 2022), which can be expected to facilitate more careful, precise, and prolonged thinking, as well as attentional engagement and willingness to expend effort. All these positive effects can be linked to the improvement of technology, since the behaviors required for advanced technological performance typically involve effort, discomfort, and sustained attention.

## Profanity and disorders

This link between derogatory language and motor skills is supported by evidence from disorders. In general, children with language impairment exhibit motor problems (Wu et al., 2021), with this comorbidity pointing to a domain-general deficit, seemingly in procedural memory abilities (Sanjeevan and Mainela-Arnold, 2019). More specifically, in Tourette's syndrome (TS), affected people show involuntary derogatory speech (Van Lancker and Cummings, 1999) and involuntary movements (and possibly, deficits in voluntary motor behavior too) (Kalsi et al., 2015). TS is not merely a disorder of speech and movement in isolation but results from a broader dysregulation of cortical-striatal pathways contributing to motor planning, procedural learning, and inhibitory control (Singer, 2005; Ganos et al., 2013). Significantly, people with TS also exhibit abnormally high level of reactive aggression (Chen et al., 2013).

## Discussion: swearing as a facilitator of technological advancement

HSD has been hypothesized to contribute to the sophistication of human culture, including our technology. This effect has been largely explained in terms of the enhancement of our prosocial behavior, which facilitated cooperation and ultimately cumulative cultural learning. The advent of more complex languages, to which HSD also contributed, helped do this too, since their larger vocabularies and more elaborated grammars are more efficient for transmitting technological knowledge and know-hows which serve as the scaffolding of subsequent advancements. Nonetheless, the emergence of complex languages, specifically, and the potentiation of HSD, more generally, also changed our brains. A key target of such changes was the subcortical-cortical network supporting procedural memory abilities. These abilities are necessary for the rule-governed aspects of language, but also of motor behavior. Achieving a more sophisticated technology does not only demand innovation and exact transmission of procedures, but also refined motor skills, particularly manual dexterity. In this paper, we have hypothesized that increased HSD might have improved our motor abilities via its effect on the brain networks supporting language, specifically procedural memory. In a nutshell, the more sophisticated language as we tamed our reactive agression, the more refined our motor abilities. Since swear words (mostly compound words) are hypothesized to have emerged early during language evolution, swearing can be viewed as the earliest instance of such a feedback loop between language (and HSD) and motor behavior. However, because swearing has other positive outcomes as well, our swearing behavior might have also potentiated our motor skills (and ultimately, our technological abilities) indirectly. Hence, for instance, fine motor skills typically require sustained effort throughout a task to achieve meaningful results. Because, as discussed above, swearing can shift attention and engagement (i.e., psychological flow), rise self-confidence, and alleviate pain, increased swearing behavior may have facilitated persistence in effortful, frustrating, and painful work, as involved in advanced technological tasks such as flintknapping. Even more, as reactive aggression decreased, communities would have tolerated more frequent and more robust social interaction. Swearing might have thus provided a context-specific outlet for the (down)regulation of aggression. Specifically, swearing may have started to serve as a linguistic tool for negotiating conflict, status, or interpersonal dynamics, as observed in present-day communities (Debray, 2023; Stapleton and Fägersten, 2023). In summary, by supporting both individual performance states and interpersonal functioning, swearing can be expected to have contributed, even if subtly, to better teaching, learning, and performance of technological procedural skills. Paraphrasing Freud's quotation above, our hypothesis is that the man who first flung a word of abuse at his enemy instead of a spear was the founder of technology.
